# 759C/T Variants of the Serotonin (5-HT2C) Receptor Gene and Weight Gain in Children and Adolescents in Long-Term Risperidone Treatment

**DOI:** 10.4172/2167-065x.1000110

**Published:** 2013-06-29

**Authors:** Nicole del Castillo, Bridget Zimmerman M, Billie Tyler, Vicki L Ellingrod, Chadi Calarge

**Affiliations:** 1 Department of Psychiatry, The University of Iowa Hospitals and Clinics, USA; 2 Department of Biostatistics, The University of Iowa College of Public Health, USA; 3 Department of Psychiatry, University of Iowa, USA; 4 Department of Clinical Social and Administrative Sciences, College of Pharmacy, Department of Psychiatry, School of Medicine, University of Michigan, USA; 5 Department of Psychiatry, The University of Iowa Carver College of Medicine, Psychiatry Research, USA

**Keywords:** Antipsychotic, Serotonin gene, Weight gain, Adolescents, Variants, Predictors

## Abstract

**Background:**

Great inter-individual variability exists in the susceptibility to gain weight during antipsychotic treatment. Thus, we examined whether the −759C/T variants in the promoter region of the 5HT2C receptor gene were differentially associated with weight gain in children and adolescents in long-term risperidone treatment.

**Methods:**

Medically healthy 7 to 17 year-olds, treated with risperidone for ≥ six months, were enrolled. Anthropometric measurements, laboratory tests, and treatment history were obtained upon enrollment and from medical records. The effect of the genotype on the trajectory of age-sex-adjusted weight and body mass index (BMI) z scores before and after the onset of risperidone treatment was investigated.

**Results:**

In 124 subjects (90% males, mean age: 11.8 years) treated with risperidone for a mean of 2.8 years, weight and BMI z scores significantly increased after starting risperidone. This change was similar across the two genotype groups as were changes in several cardiometabolic variables.

**Conclusion:**

In contrast to other reports, the T allele failed to confer protection against excessive weight gain or cardiometabolic abnormalities in this group of children and adolescents chronically treated with risperidone.

## Introduction

Obesity is one of the most serious public health concerns in the 21^st^ century [1]. In particular, childhood obesity tracks into adulthood [2,3], increasing the risk for a variety of chronic conditions and reducing life expectancy [4,5]. Moreover, obesity can result in psychosocial sequelae and interfere with treatment adherence [6]. Thus, the prevention and treatment of obesity are imperative, particularly in children and adolescents.

The use of second-generation antipsychotics (SGAs) in the pediatric population has dramatically increased over the last two decades [7,8] despite the potential for significant weight gain which frequently results in obesity [9–11]. This is likely due to several factors including growing evidence for efficacy in a broad range of psychiatric disorders such as schizophrenia, mood disorders, tic disorders, and disruptive behavior disorders [12,13] as well as the need to address increasingly challenging behavior in the least restrictive environments. Therefore, as the use of SGAs grows in this vulnerable population, it is necessary to better understand their effect on weight and cardiometabolic health to optimize their long-term tolerability.

SGA-induced weight gain is multifactorial, involving environmental and biological factors [14]. In fact, significant interindividual variability exists in the magnitude of weight gained during SGA treatment [15–17]. In children and adolescents, several factors have been associated with SGA-related weight gain including 1) weight at the onset of SGA treatment, 2) prior and concurrent psychotropic treatments, 3) as well as SGA dose which has been implicated in some, but not all, studies [17–23].

Furthermore, important, albeit small, adult twin studies have estimated the heritability of SGA-induced weight gain at 60 to 80% [24,25], confirming that genetic factors account for, at least, part of the susceptibility to this side effect. In fact, the serotonin 5HT2C receptor gene has been of particular interest due to its association with feeding behavior [26] and the fact that 5HT2C receptor gene knockout mice develop obesity [27]. Moreover, the propensity of antipsychotic medications to cause weight gain appears to be related to their affinity for this receptor [28]. Importantly, a C to T transversion at position −759 (-759C/T) in the promoter region of the 5HT2C receptor gene has been associated with a differential rate of gene transcription as well as obesity and diabetes in the general population [29–33], Furthermore, several studies have implicated these variants specifically in antipsychotic-related weight gain, showing a protective effect of the T allele [28,34–42].

Thus, taking advantage of a unique and well-characterized group of children and adolescents in long-term risperidone treatment, we sought to examine whether the T allele of the 5HT2C receptor gene protected against risperidone-related weight gain. Risperidone is the most commonly prescribed SGA in children and adolescents [43–47]. Notably, it is also thought to be the SGA most susceptible to the effects of various patient- and drug-specific factors regarding its potential to induce weight changes during chronic treatment [48]. This makes investigating predictors of weight gain with this drug particularly promising.

## Methods

### Subjects

This study has been previously described [20,49]. Briefly, 7 to 17 year-old patients treated with risperidone for six months or more were enrolled, irrespective of their primary psychiatric diagnosis or indication for risperidone. Concurrent treatment at enrollment with additional psychotropics, but not with other antipsychotics, was allowed. Participants with neurological or medical conditions that could confound the metabolic or hormonal assessments were excluded as were pregnant females and those receiving hormonal contraception.

### Procedures

This study was approved by the local Institutional Review Board. Assent was obtained from children ≤ 14 years old and written consent from adolescents and all parents or legal guardians.

Race and ethnicity were based on self-report. Using the medical record, the indication for risperidone, the start and stop dates of each psychotropic, and changes in the dosage and formulation were recorded [20]. This documentation was confirmed by a physician and reflected deviations from the prescribed treatments. All dosages of psychostimulants were expressed in methylphenidate-equivalent for amphetamines (x 2) [50].

Upon enrollment, triceps and subscapular skinfold thickness was measured with a Lange skinfold caliper to the nearest 0.1 mm by one of two research dietitians (inter-rater agreement ICC>95%, n=16) [20]. The average of two measurements was used. Height was measured to the nearest 0.1 cm using a stadiometer (Holtain Ltd., UK) and weight was recorded to the nearest 0.1 kg using a digital scale (Scaletronix, Wheaton, IL), while wearing indoor clothes without shoes. In addition, all height and weight measurements were extracted from the medical record. When both height and weight were available, body mass index (BMI, kg/m^2^) was computed. Importantly, measurements collected during clinical and research encounters falling within a month of each other were highly correlated [mean interval between visits=16 days (sd=9) for height (n=69) and 17 days (sd=9) for weight (n=97)]. The intra-class correlations were all above 0.97 (95% confidence intervals [CI] ranging between 0.96–0.99) for unadjusted height and weight as well as for their sex- and age-adjusted z scores.

Pubertal stage was evaluated by a physician and the participants, with parental help when necessary. Interrater agreement between the physician and self-rating was high (weighted kappa=0.81, 95% confidence interval (C.I.)=0.74–0.88).

A best-estimate diagnosis, following the Diagnostic and Statistical Manual of Mental Disorders (DSM-IV-TR) [51], was generated based on a review of the psychiatric record often supplemented by a brief clinical interview, a standardized interview of the parent using the NIMH Diagnostic Interview Schedule for Children (DISC-IV) [52], and the Child Behavior Checklist [53].

A morning blood sample was obtained, after at least a 9-hr overnight fast, to measure glucose (Roche Diagnostics, Indianapolis, IN), total insulin (Diagnostic Products, Los Angeles, CA), total cholesterol, HDL cholesterol (HDL), and triglycerides (Roche Diagnostics, Indianapolis, IN). LDL cholesterol was estimated following Friedewald’s equation [54]. Participants who were not fasting or whose fasting status was missing were excluded from the analyses related to the laboratory measures.

DNA was extracted from a whole blood sample using a Purgene kit (Qiagen, Valencia California). After the samples were processed, they underwent spectrophotometry to establish purity and yield and were then frozen at −80°C. Polymerase chain reaction (PCR) and sequencing primers for the −759C/T variants (dbSNP rs3813929) were designed using Pyrosequencing SNP Primer Design Version 1.01 software (http://www.pyrosequencing.com). PCR products were visualized by electrophoresis on 1.8% agarose gels stained with ethidium bromide before Pyrosequencing. Genotyping was done with Pyrosequencing™ Technology [55]. Details regarding this assay are available upon request.

### Statistical analysis

Participants with missing baseline weight (or BMI), i.e., one obtained within one month before the initiation of risperidone, and those exposed to antipsychotics other than risperidone prior to starting risperidone were excluded from the analysis. If, after starting it, a participant discontinued risperidone or received an additional antipsychotic, all subsequent anthropometric measurements were excluded. Also, observations collected before age 2 years were excluded.

In order to account for children’s natural growth, age-sex-specific z scores for weight and BMI were calculated [56]. Blood pressure measurements were also converted into age-sex-height-adjusted z scores [9]. Body fat was estimated using skin-fold thickness measurements following Slaughter et al. [57] and the homeostasis model assessment insulin resistance index (HOMA-IR) was estimated as: [insulin (µUI/ml)×glucose (mg/dl)]/405 [58].

The sample was divided based on the presence of the T allele (i.e., T(+) [n=18] versus T(−) [n=106] groups). Differences across the two genotype groups were compared using the Student t-test for continuous variables and Fisher’s exact test for categorical ones. When necessary, analysis of covariance (ANCOVA) was used to adjust for age and sex.

In order to explore the genotype effect on the trajectory of weight gain during risperidone treatment, a random coefficient mixed regression model was fitted, having weight (or BMI) z score as the dependent variable and baseline weight (or BMI) z score, the weight-adjusted daily dose of methylphenidate (mg/kg/day), genotype group, and genotype group × risperidone treatment status × time 3-way interaction effects as predictor variables. The model included fixed and random effects for intercept and slope (i.e., time), to represent the mean curve and the random variation of each child’s curve from the mean curve, respectively. Since we anticipated that weight gain from risperidone will plateau, time was modeled as a linear and quadratic effect. Consequently, we required a minimum of three weight (or BMI) measurements before or three after risperidone initiation in order for a participant to contribute to the analysis.

All the statistical tests were two-tailed, with statistical significance set at α=0.05, and performed using SAS version 9.2 for Windows (SAS Institute Inc, Cary, North Carolina).

## Results

### Participants

As shown in Table 1, the majority of the participants included in the analysis were males (n=112, 90%) and non-Hispanic Caucasian (n=101, 82%). The T allele carriers were slightly older and more sexually mature than the T(−) genotype group.

Table 2 summarizes the psychiatric and treatment characteristics of the sample. Externalizing disorders were prevalent and most participants received risperidone for aggression or irritability (90%). Polypharmacy was common with psychostimulants (73%), selective serotonin reuptake inhibitors (51%), and α_2_-agonists (31%) being the most concurrently prescribed agents.

After adjusting for age and sex, the two genotype groups did not differ on any clinical characteristic except for anxiety disorders being more common among the T(−) group.

### Measurements at study enrollment

Table 3 reports the anthropometric and cardiometabolic measurements of the sample. Over the course of risperidone treatment, weight and BMI z scores increased significantly but this was comparable across the two genotype groups. Again, after controlling for age and sex, none of the cardiometabolic variables differed across the two genotype groups.

### Trajectory of weight gain

**Figure 1:**
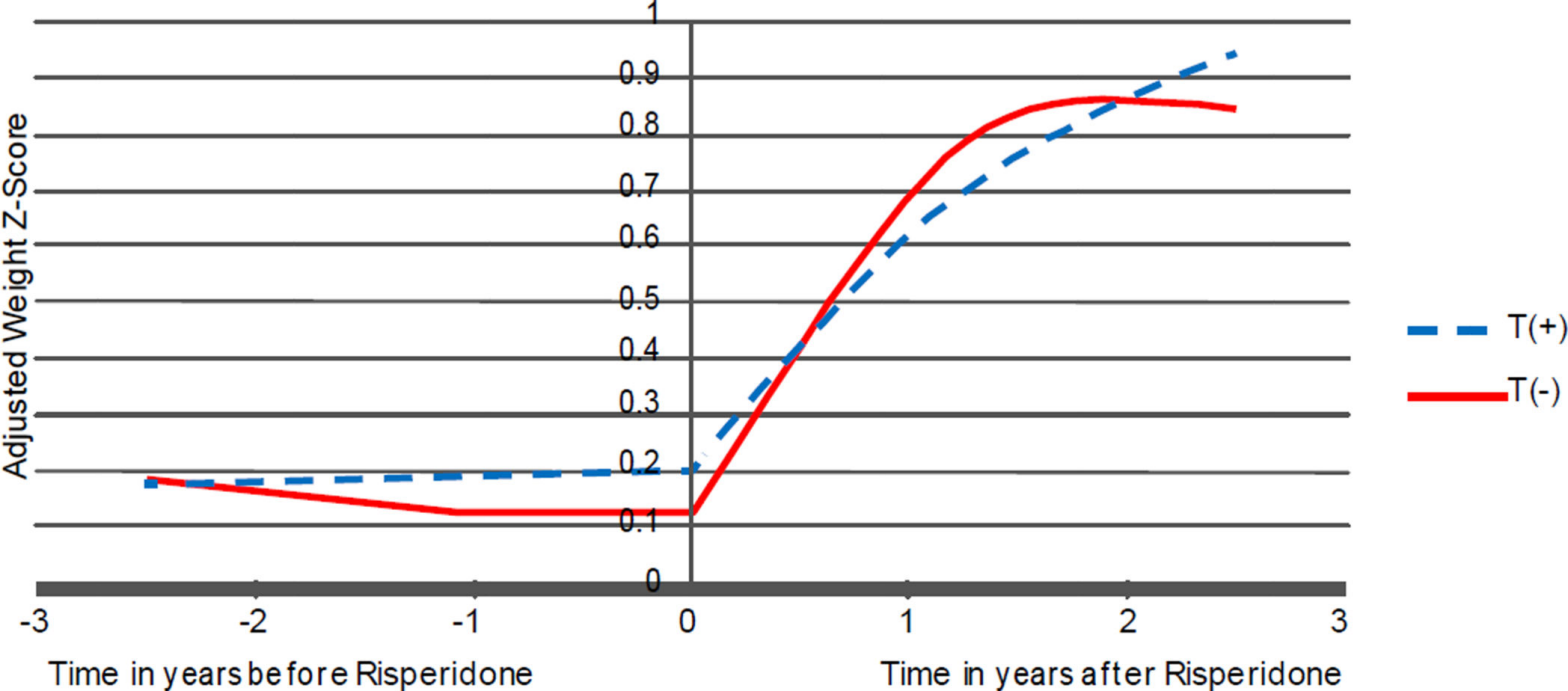
Estimated Weight Z-Score over time for the two 5HT2C Genotype Groups.

Using mixed model regression analysis, after controlling for baseline weight z score (β=0.953, p<0.0001) and the weight-adjusted daily dose of methylphenidate (β=−0.173, p<0.0001), there was a significant genotype by risperidone treatment by time 3-way interaction effect (p<0.0001). In fact, the trajectories of change in weight z score before and after risperidone was started were significantly different (p<0.0002). As shown in Figure 1, there was no significant change in weight z score before risperidone was started. In contrast, after the onset of risperidone treatment, weight z score increased significantly. However, the trajectories across the two genotype groups were comparable both before as well as after risperidone was started, for both the linear (p>0.4) as well as the quadratic (p>0.05) effects of time.

**Figure 2:**
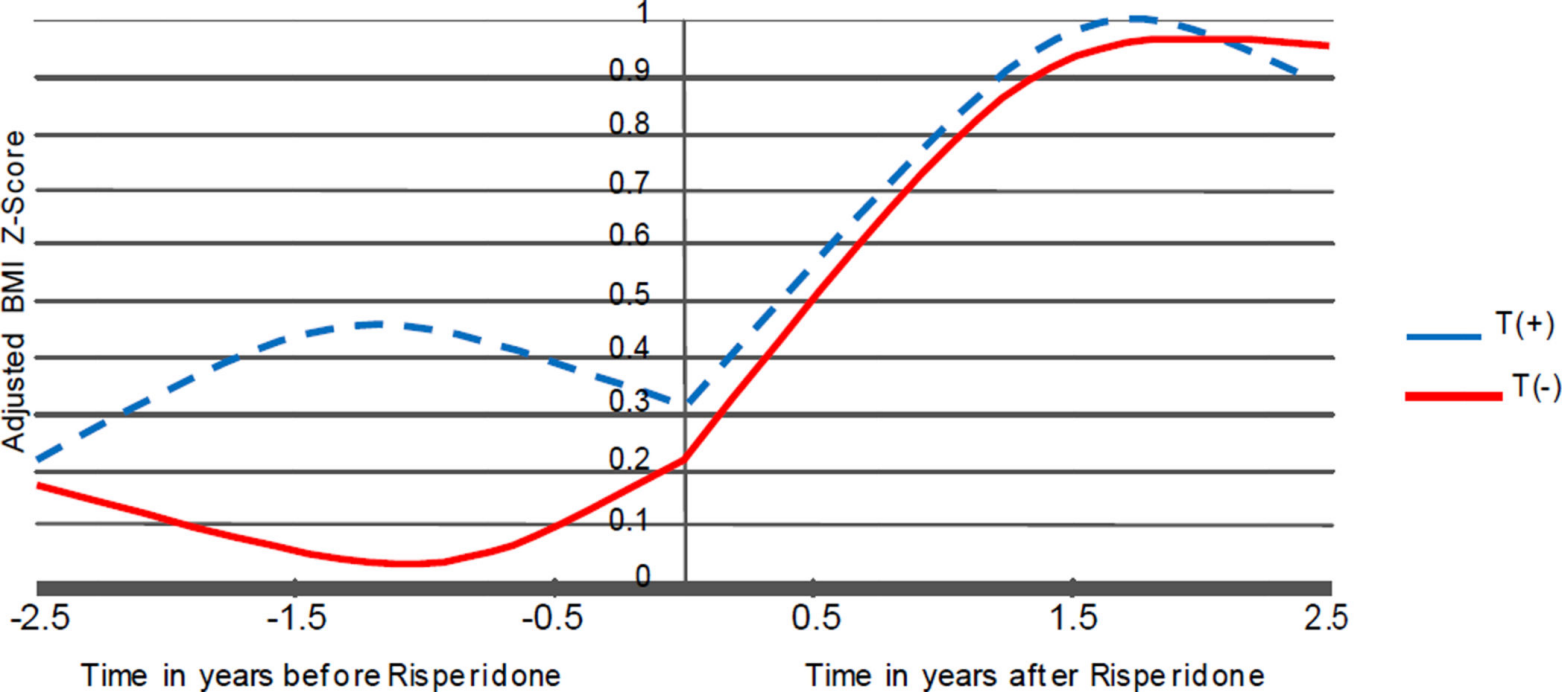
Estimated BMI Z-Score over time for the two 5HT2C Genotype Groups.

Similarly, after controlling for baseline BMI z score (β=0.867, p<0.0001) and the weight-adjusted daily dose of methylphenidate (β=− 0.200, p<0.0001) (Figure 2), the genotype by risperidone treatment by time 3-way interaction effect was significant (p<0.0001). Again, this reflected the fact that BMI z score increased significantly after risperidone was started (p<0.0001). Interestingly, the trajectories of BMI z score was different across the two genotype groups before risperidone was started, with the T(+) allele showing a slight increase in BMI z score (p<0.03). However, the trajectories were not different after risperidone was started for either the linear (p>0.8) or the quadratic (p>0.9) effect of time.

When the analyses were repeated after restricting the sample to non-Hispanic Caucasians, the findings did not change appreciably.

## Discussion

To our knowledge, this is the first pediatric study to evaluate the potential role of the −759C/T variants in the promoter region of the 5HT2C receptor gene in weight gain following long-term treatment with SGAs. In contrast to our hypothesis and several published studies [17,28,34–42], the T allele was neither associated with less weight gain nor with a reduced risk for cardiometabolic abnormalities.

For decades, the role of serotonin in feeding behavior has been of interest with significant advances made delineating the specific pathways involved [59,60]. This has also led to research investigating the role of specific subtypes of serotonin receptors. In particular, the 5HT2C receptor has been studied in regards to food consumption and satiety [60]. For instance, 5HT2C receptor gene knock-out mice overeat, are obese, and exhibit increased serum glucose, leptin, and insulin concentrations [27]. In addition, 5HT2C receptor antagonists have also been found to prevent or delay the onset of satiety [18]. Of interest, several SGAs exhibit a high affinity for the 5HT2C receptor in contrast to first-generation antipsychotics [18,28]. In fact, this affinity appears to be correlated with the propensity of the drugs to cause weight gain [28]. As a result, several studies have explored whether functional variants of the 5HT2C receptor gene are implicated in the risk for antipsychotic-related weight gain. Specifically, the −759C/T variants have been of interest since they are relatively common in the general population. Moreover, the T allele has been associated with increased basal expression of the 5HT2C receptor [33], although this finding has not been consistent [61]. Nonetheless, this focus has made the T allele one of the genetic variants most frequently investigated in antipsychotic-related weight gain [62], with several studies finding it to be protective compared to the C allele [28,34–42].

The protective potential of the T allele has also been recently confirmed in a small trial in children with autistic disorder receiving risperidone for 8 weeks [17]. It is unclear why our findings differed from that study but several reasons could explain the discrepancy. We included a heterogeneous clinical group, in polypharmacy, treated with risperidone for an average of nearly three years. In contrast, the study by Hoekstra et al. [17] was restricted to youth with autistic disorder, treated with risperidone for 8 weeks, with only 25% receiving another psychotropic (namely psychostimulants). Similarly, it is likely that differences in study design explain the discrepancy between studies reporting a protective effect of the T alleles [28,34–42] and those not [63–70]. In fact, factors thought to influence this association include prior antipsychotic treatment status, duration of treatment (< vs. >3 months), the particular drug investigated, race, and sample size [62].

Our participants had undergone treatment with risperidone for nearly three years. Others have shown that acute and chronic antipsychotic-induced weight gain may be linked to different genetic risk factors [71]. Leptin and serotonin both reduce food intake and promote energy expenditure [72]. A variant in the promoter region (−2548A/G) of the leptin gene has been associated with obesity [42]. In antipsychotic-treated patients, Templeman et al. [42] found that the A allele protected against excessive weight gain after 9 months of treatment but not acutely (i.e., after 6 and 12 weeks of treatment). In contrast, carriers of the T allele of the 5HT2C gene, at position −759, gained significantly less weight than those without this allele, following both acute and extended treatment [42]. Interestingly, the −759C/T genotype was significantly associated with pre-treatment plasma leptin levels [42]. In an earlier study, using an overlapping sample, we found that variants of the leptin gene predicted weight gain from risperidone [73]. How the −759C/T variants of the 5-HT2C receptor gene might interact with the −2548A/G variants would be critical to explore since the contribution of any single gene to a complex phenotype, like antipsychotic-related weight gain, is probably limited [74].

There were several limitations to this study. First, unlike most other studies, our sample consisted of a heterogeneous clinical group receiving psychotropic polypharmacy. However, SGAs appear to cause comparable weight gain across most pediatric psychiatric disorders (except eating disorders perhaps) [11,75,76]. In fact, the magnitude of weight gain we found is similar to what others have reported [21]. Some demographic and clinical characteristics differed across the two genotype groups but these may reflect the relatively small sample size of the T allele carriers and the number of statistical comparisons conducted. Nonetheless, we accounted in the analyses for pertinent variables (e.g., age). Another shortcoming is that, except for psychostimulants, we failed to account for treatment with other psychotropics that may alter weight. Thirdly, although risperidone is the most commonly prescribed antipsychotic in children [43–47], it is unclear how our findings might generalize to other antipsychotics. In addition, most anthropometric measurements were extracted from the medical record. However, as we note earlier and as shown by others [77], the measurements collected during clinical encounters were highly correlated with those obtained following standard research procedures. Finally, our sample consisted primarily of non-Hispanic Caucasian males, reflecting the local racial/ethnic composition and national trends of antipsychotic use [12,78]. Thus, future studies should better represent females and participants from a diverse background, while considering that allele frequency might vary across racial/ethnic groups [62,79].

## Conclusions

In summary, in our sample of children and adolescents in chronic risperidone treatment, the −759C/T variants failed to differentially predict weight gain. The inconsistency in the literature suggests that further research is needed before pharmacogenetics can fulfill its promise to guide clinicians in individualizing antipsychotic treatment.

## Figures and Tables

**Table 1:** Demographic Characteristics of the Sample Divided Based on T Allele Carrier State.

Characteristicsc	All Subjects (N=124)	T(+) Genotypes (N=18)	T(−) Genotype (N=106)	Statistical Analysis	P
**Male, n (%)**	112 (90)	15 (83)	97 (92)	Fisher’s Exact	0.4
**Age, mean ± SD, y**	11.8 ± 2.8	13.1 ± 2.6	11.6 ± 2.8	t= 2.20, df=122	<0.03
**Race/Ethnicity, % Caucasian/African American/Hispanic/Other**	82/14/3/1 [n=123]	13/0/1.5/0	69/14/1.5/1 [n=105]	Fisher’s Exact	>0.06
**Pubertal Status, % at Tanner stage I, II, III, IV, V**	34/18/17/19/11 [n=122]	2/2/2/6/3	32/16/15/13/8 [n=104]	Fisher’s Exact	<0.05

**Table 2:** Psychopathology and Medication history in the Sample Divided Based on T Allele Carrier State.

	All Subjects (N=124)	T(+) Genotype (N=18)	T(−) Genotype (N=106)	Statistical Analysis	P
**Psychopathology**					
ADHD, n (%)	110 (89)	14 (78)	96 (91)	Fisher’s Exact	>0.1
DBD, n (%)	112 (90)	17 (94)	95 (90)	Fisher’s Exact	1.0
Anxiety Disorder, n (%)	40 (32)	2 (11)	38 (36)	Fisher’s Exact	**<*0.05***
Tic Disorder, n (%)	27 (22)	5 (28)	22 (21)	Fisher’s Exact	>0.5
PDD, n (%)	19 (15)	3 (17)	16 (15)	Fisher’s Exact	1.0
Depressive Disorder, n (%)	11 (9)	1 (6)	10 (9)	Fisher’s Exact	1.0
Psychosis, n (%)	1 (1)	0	1 (1)	Fisher’s Exact	1.0
**Pharmacotherapy**					
Risperidone Dose, (mg/kg/d), mean ± sd	0.03 ± 0.02	0.03 ± 0.03	0.03 ± 0.02	t= −0.55	>0.6
Risperidone Duration, (years), mean ± se	2.8 ± 2.0	3.1 (0.5)^a^	2.7 (0.3)^a^	t=0.7	>0.5
Psychostimulants, n (%)	90 (73)	10 (56)	80 (75)	Fisher’s Exact	>0.09
MPH Dose, (mg/kg/d), *mean* ± sd	1.3 ± 0.7 [n=90]	1.1 ± 0.4 [n=10]	1.4 ± 0.69 [n=80]	t=−1.24	>0.2
MPH Treatment Duration, yrs, mean ± *se*	5.2 ± 2.8	5.4 ± 0.6^a^	4.8 ± 0.3^a^	t=1.0	>0.3
SSRIs, n (%)	63 (51)	8 (44)	55 (52)	Fisher’s Exact	>0.6
α_2_-agonsits, n (%)‡	39 (31)	7 (39)	32 (30)	Fisher’s Exact	>0.6

a Results for ANCOVA of the value across the two genotype groups, controlling for age and sex

ADHD: Attention Deficit Hyperactivity Disorder; DBD: Disruptive Behavior Disorder; PDD: Pervasive Developmental Disorder; MPH: Methylphenidate; SSRIs: Selective Serotonin Reuptake Inhibitors

**Table 3:** Mean ± sd of Anthropometric and Cardiometabolic Variables in the Sample Overall and Divided Based on T Allele Carrier State.

Variable	All Subjects (N=124)	T(+) Genotype (N=18)	T(-) Genotype (N=106)	Statistical Analysis	P Value
**Baseline Weight z score**	0.1 ± 1.0	0.3 ± 1.2	0.07 ± 0.96	t=1.0	>0.2
**Change in Weight z Score**	0.5 ± 0.7	0.46 ± 0.8	0.5 ± 0.7	t=−0.37	>0.7
**Baseline BMI z score**	0.14 ± 1.0 [n=121]	0.04 ± 1.3 [n=17]	0.15 ± 1.0 [n=104]	t=−0.42	>0.7
**Change in BMI z Score**	0.5 ± 0.8 [n=121]	0.6 ± 1.02 [n=17]	0.5 ± 0.8 [n=104]	t=0.39	>0.7
**Waist Circumference, (cm)**	72.2 ± 13.3 [n=109]	76.4 ± 3.0 [n=17]^a^	72.4 ± 1.9 [n=92]^a^	1.28	>0.2
**Percent Body Fat**	21.2 ± 10 [n=104]	25 ± 2.7 [n=17]^a^	23 ± 1.7 [n=88]^a^	t=0.8	>0.4
**Total Cholesterol, (mg/dl)**	156.0 ± 25.0 [n=100]	144.1 ± 7.4 [n=15]^a^	156.6 ± 5.2 [n=85]^a^	t=−1.8	>0.07
**HDL Cholesterol, (mg/dl)**	57.3 ± 12.6 [n=101]	60.5 ± 3.5 [n=15]^a^	60.0 ± 2.5 [n=86]^a^	t=0.1	>0.9
**LDL Cholesterol, (mg/dl)**	87.1 ± 21.8 [n=101]	76.5 ± 6.4 [n=15]^a^	84.5 ± 4.5 [n=86]^a^	t=−1.3	>0.2
**Triglycerides, (mg/dl)**	56.7 ± 32.1 [n=100]	45.4 ± 8.9 [n=15]^a^	55.7 ± 6.2 [n=85]^a^	t=−1.2	>0.2
**Glucose, (mg/dl)**	89.4 ± 8.3 [n=101]	88.6 ± 2.5 [n=15]^a^	88.9 ± 1.7 [n=86]^a^	t=−0.1	>0.9
**Total Insulin, (µlU/ml)**	7.2 ± 5.04 [n=90]	7.2 ± 5.04 [n=90]	8.0 ± 1.0 [n=77]a	t=0.5	>0.6
**HOMA_IR**	1.6 ± 1.1 [n=100]	1.9 ± 0.3 [n=15]^a^	1.8 ± 0.2 [n=85]^a^	t=0.5	>0.6

a ANCOVA controlling for age and sex.

The cardiometabolic results are reported only for individuals who had fasted for ≥ 9 hours.
